# Respiratory Syncytial Virus Disease Burden and Evidence Gaps in Australia Before Immunisation Introduction: A Retrospective Population‐Based Study Using 2016–2019 Hospitalisation and 2023 Laboratory Surveillance Data

**DOI:** 10.5694/mja2.70289

**Published:** 2026-09-17

**Authors:** Chisato Imai‐Boulter, Jean Li‐Kim‐Moy, Clayton Chiu, Sanjay Jayasinghe, Kristine Macartney

**Affiliations:** ^1^ National Centre for Immunisation Research and Surveillance Sydney New South Wales Australia; ^2^ Faculty of Medicine and Health Children's Hospital at Westmead Clinical School, University of Sydney Sydney New South Wales Australia; ^3^ Department of Global Health Policy Graduate School of Medicine, University of Tokyo Tokyo Japan

**Keywords:** Epidemiology, Gerontology, Morbidity, Mortality, Paediatrics, Public health, Respiratory tract infections, Surveillance, Vaccination, Vaccine preventable disease

## Abstract

This study evaluated Australia's national respiratory syncytial virus (RSV) burden and highlighted key data gaps. Hospitalisations (2016–2019) peaked in infants aged < 3 months (4014/100,000) and adults aged ≥ 65 years (122/100,000), nearly doubling over the period. Unspecified causes comprised ≈55% of bronchiolitis hospitalisations in children aged < 24 months. Laboratory notifications (2023) peaked at 6–11 months (7778/100,000). Real‐time data integration addressing case‐ascertainment and diagnostic testing frequency is essential for accurate burden estimates and future immunisation strategies.

## 1 Introduction

Australia's respiratory syncytial virus (RSV) disease landscape has shifted since recent immunisation program rollouts targeting infants and older adults, including maternal immunisation for infant protection. However, national burden assessments using data since 2015 remain limited [1]. This study provides pre‐implementation national RSV burden estimates using existing datasets, highlighting data improvements needed for effective post‐implementation monitoring.

## 2 Methods

RSV‐related hospitalisations and in‐hospital mortality for 2016–2019, before COVID‐19 pandemic‐related disruptions [2, 3], were assessed using the Australian Institute of Health and Welfare (AIHW) National Hospital Morbidity Database. Cases were identified using International Classification of Diseases, Tenth Revision, Australian Modification (ICD‐10‐AM) codes (Table S1). Seasonality was defined as April to September (in‐season) and October to March (inter‐season) [3, 4].

Among children aged < 24 months with principal diagnosis of acute bronchiolitis, we examined the proportions of RSV‐specific (J21.0) and unspecified (J21.9) bronchiolitis, the latter commonly coded in untested children (Table S1) [5].

Laboratory‐confirmed RSV notifications (2023) were sourced from the National Notifiable Diseases Surveillance System (NNDSS), as national reporting was incomplete before September 2022.

Rates were calculated using Australian Bureau of Statistics annual population estimates as denominators. Sensitivity analyses were performed on 2020–2022 hospitalisations. Methods S1 provides additional details.

We reported our study according to the STROBE statement for reporting cohort studies (Table S2) [6].

## 3 Results

In 2016–2019, there were 70,298 RSV‐related hospitalisations, with 51.7% in children aged < 24 months. Rate peaked in infants aged < 3 months (4014 per 100,000 population), declining with age but rising markedly from ≥ 75 years (Table 1; Table S3).

**TABLE 1:** Respiratory syncytial virus (RSV)‐related hospitalisation rates and in‐hospital case fatality rate (CFR) by age group, 2016–2019.

	Total hospitalisations *n* (%)	Hospitalisation rate per 100,000 population^a^	In‐hospital CFR % (95% CI)^b^
**Age groups**			
< 3 months	12,343 (17.6%)	4014	< 0.1% (0.0%–0.1%)
3–5 months	6694 (9.5%)	2177	< 0.1% (0.0%–0.1%)
6–11 months	7961 (11.3%)	1294	0.1% (0.0%–0.2%)
12–23 months	9379 (13.3%)	755	< 0.1% (0.0%–0.1%)
24–59 months	5573 (7.9%)	147	0.1% (0.0%–0.2%)
5–19 years	1908 (2.7%)	10	0.7% (0.4%–1.2%)
20–49 years	2857 (4.1%)	7	1.0% (0.7%–1.4%)
50–59 years	2527 (3.6%)	21	2.0% (1.4%–2.6%)
60–64 years	2059 (2.9%)	38	2.0% (1.5%–2.8%)
65–69 years^c^	2568 (3.7%)	53	3.1% (2.4%–3.8%)
70–74 years^c^	3166 (4.5%)	81	3.4% (2.8%–4.1%)
75–79 years^c^	3326 (4.7%)	121	3.3% (2.7%–4.0%)
≥ 80 years^c^	9690 (13.8%)	249	4.6% (4.2%–5.0%)
**Aggregated age groups**			
< 6 months	19,037 (27.1%)	3095	< 0.1% (0.0%–0.1%)
< 12 months	26,998 (38.4%)	2195	< 0.1% (0.0%–0.1%)
< 24 months	36,377 (51.7%)	1472	< 0.1% (0.0%–0.1%)
≥ 60 years^d^	21,056 (30.0%)	101	3.8% (3.6%–4.2%)
≥ 65 years^d^	18,997 (27.0%)	123	4.1% (3.8%–4.4%)
All ages^d^	70,298 (100.0%)	71	1.3% (1.2%–1.4%)

Abbreviation: CI, confidence interval.

^a^ Annual rates from 2016 to 2019 increased across all age groups, likely due to systemic changes (i.e., case‐ascertainment) rather than true epidemiological changes; therefore, year‐specific rates, rather than CIs, are provided in Table S3.

^b^ 95% CIs for in‐hospital CFR (%) were calculated using the Clopper–Pearson exact method.

^c^ Age‐specific analysis for people aged ≥ 65 years includes only people who did not identify as Aboriginal or Torres Strait Islander. Age‐stratified hospitalisation data for Aboriginal and Torres Strait Islander peoples aged ≥ 65 years were not supplied to the authors due to small numbers and to protect against individual identification.

^d^ Aboriginal and Torres Strait Islander peoples aged ≥ 65 years are included in these aggregated age groups.

Among children aged < 24 months, in‐season and inter‐season hospitalisation rates were 1149 and 322 per 100,000 population, respectively (Table S4). Although inter‐season rates were ≈20% of annual rates across age groups, for children aged < 24 months they exceeded annual rates in all older age groups.

By 2019, hospitalisation rates were 1.2 and 1.9 times higher than in 2016 for those aged < 24 months and ≥ 65 years, respectively.

Among children aged < 24 months, unspecified causes accounted for ≈55% of hospitalisations for acute bronchiolitis. Of the hospitalisations with a specified pathogen, RSV‐specific codes comprised ≈70% (Figure S1).

The overall in‐hospital case fatality rate (CFR) increased with age, from < 0.1% in infants aged < 6 months to 4.1% in adults aged ≥ 65 years (Table 1).

**FIGURE 1:**
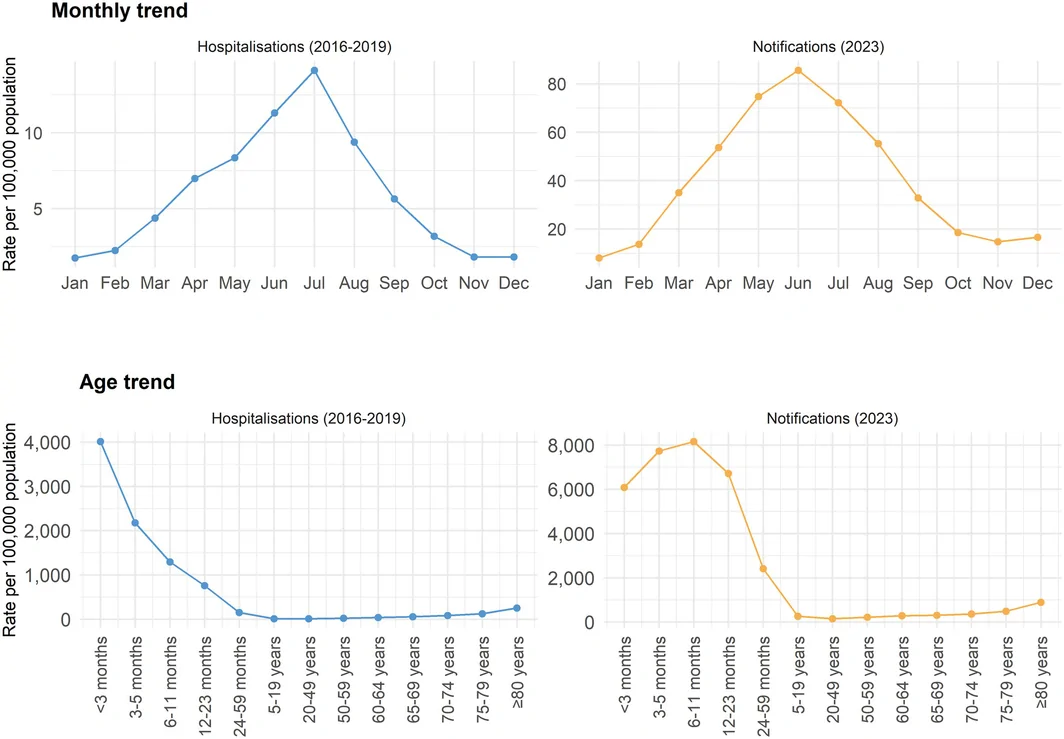
Monthly and age trends of respiratory syncytial virus (RSV)‐related hospitalisations in 2016–2019 and laboratory‐confirmed RSV notifications in 2023.

In 2023, 128,103 RSV notifications were recorded nationally, peaking in July with seasonality mirroring hospitalisations (Figure 1). Notification rates peaked in older infants aged 6–11 months (7778 per 100,000 population) and remained elevated for ages < 24 months (Figure 1).

Sensitivity analyses of 2020–2022 hospitalisation rates were consistent with 2016–2019 data, peaking at age < 3 months and declining with increasing age (Figure S2).

## 4 Discussion

Children aged < 24 months experience substantial, year‐round burden, whereas older adults (aged ≥ 75 years) face high hospitalisation rates and the highest in‐hospital CFRs, underscoring the significance of the funded immunisation programs to protect these age groups. Although hospitalisation and notification data are essential for assessing RSV burden, quality and completeness limitations constrain their utility.

ICD‐10‐AM‐coded hospitalisations likely underestimate RSV burden due to limited diagnostic sensitivity [7]. The high proportion of unspecified bronchiolitis observed among young children suggests a portion of RSV‐associated bronchiolitis remains under‐diagnosed. Despite expanded post‐COVID‐19 pandemic multiplex respiratory virus testing, under‐ascertainment likely persists, especially in older adults with historically lower testing rates [8]. Between 2016 and 2019, older‐adult hospitalisation rates nearly doubled—a trend unmatched in young children—suggesting that improved case ascertainment was more likely the cause rather than a true incidence increase.

Mortality estimates also indicate under‐ascertainment: the observed in‐hospital CFR for adults aged ≥ 65 years was below global estimates (6.1%; 95% CI, 3.3–11.0) [8]. Furthermore, our preliminary examination of NNDSS and national mortality records revealed minimal RSV‐related deaths, precluding reliable mortality analysis.

Missing testing denominators make interpreting NNDSS data challenging. Although 2016–2019 hospitalisation rates peaked at age < 3 months, 2023 notification rates peaked at age 6–11 months and remained elevated through age 24 months. This likely reflects differing age distributions of severe and milder disease (included among notified cases) rather than post‐COVID‐19 pandemic testing artefacts, given similar hospitalisation age trends between 2016–2019 and 2020–2022. However, primary drivers remain uncertain without underlying testing frequencies to adjust for these age trends.

Reliance on existing standalone datasets limits the reliability and interpretability of RSV burden estimates, reinforcing the need for an integrated data system. Although the AIHW National Health Data Hub links immunisation, hospitalisation and mortality data [9], which could facilitate improved burden assessments and programme‐impact evaluations, it currently excludes NNDSS data. Incorporating these laboratory surveillance data with testing denominators is essential to distinguish true epidemiological changes from variations in testing frequency. Establishing real‐time linkage would further enable the system to accurately capture a broader spectrum of outcomes, including non‐hospitalised cases and out‐of‐hospital deaths, ultimately strengthening disease monitoring and informing timely immunisation strategies in Australia.

## Author Contributions

**Chisato Imai‐Boulter:** conceptualisation, methodology, statistical analysis, interpretation of data, writing the original draft and writing – review and edit. **Jean Li‐Kim‐Moy:** conceptualisation, interpretation of data, supervision and writing – review and edit. **Clayton Chiu:** conceptualisation, methodology, interpretation of data, supervision and writing – review and edit. **Sanjay Jayasinghe:** data curation, interpretation of data and writing – review and edit. **Kristine Macartney:** interpretation of data, supervision and writing – review and edit.

## Funding

The authors have nothing to report.

## Disclosure

Not commissioned; externally peer reviewed.

## Conflicts of Interest

All authors are employees of the National Centre for Immunisation Research and Surveillance, which receives funding from the Australian Government Department of Health, Disability and Ageing and the Australian Centre for Disease Control for undertaking scientific research and technical work that informs immunisation policies and programs. This work represents the views of the authors only and does not necessarily represent the views of the Australian Government.

## Supporting information


**Table S1:** ICD‐10‐AM codes used to define hospitalisations associated with RSV and acute bronchiolitis.
**Table S2:** STROBE Statement—Checklist of items that should be included in reports of cohort studies.
**Table S3:** Age‐specific RSV‐related hospitalisation rates by year.
**Table S4:** In‐season and inter‐season RSV‐related hospitalisation rates and proportions of annual rate, 2016–2019.
**Figure S1:** Hospitalisations with a principal diagnosis of acute bronchiolitis (J21) by causative organisms, 2016–2019.
**Figure S2:** RSV‐coded hospitalisation rates: 2016–2019 (pre‐COVID‐19) and 2020–2022.

## Data Availability

The de‐identified data used in this study are not publicly available and cannot be shared, as permission to release the data has not been granted by the data custodians.
