# Supplementary material for: Respiratory Syncytial Virus Disease Burden and Evidence Gaps in Australia Before Immunisation Introduction: A Retrospective Population‐Based Study Using 2016–2019 Hospitalisation and 2023 Laboratory Surveillance Data
Source: Med J Aust. 2026 Sep 17;224(9):e70289. doi: 10.5694/mja2.70289 (PMC13585565; doi:10.5694/mja2.70289)

Supporting Information

Supplementary material

This appendix was part of the submitted manuscript and has been peer reviewed.
It is posted as supplied by the authors.

Appendix to: C. Imai-Boulter, J. Li-Kim-Moy, C. Chiu, et al. Respiratory Syncytial Virus Disease Burden and Evidence Gaps in Australia Before Immunisation Introduction: A Retrospective Population-Based Study Using 2016–2019 Hospitalisation and 2023 Laboratory Surveillance Data. *Medical Journal of Australia* 2026; doi: 10.5694/mja2.70289.

Table S1. ICD-10-AM codes used to define hospitalisations associated with RSV and acute bronchiolitis.

Outcomes	Diagnosis fields	ICD-10-AM	Age group
RSV-related hospitalisations (Primary analysis of 2016-2019 hospitalisation rates)	Principal and additional diagnoses	• J12.1: RSV pneumonia<br>• J20.5: RSV bronchitis<br>• J21.0: Acute bronchiolitis due to RSV<br>• B97.4: RSV as the cause of diseases classified elsewhere	All age groups
Acute bronchiolitis hospitalisations (Secondary analysis)	Principal diagnosis	The ICD-10 system classifies J21 (acute bronchiolitis) into four etiology-specific subclasses:<br>• J21.0: Acute bronchiolitis due to RSV<br>• J21.1: Acute bronchiolitis due to human metapneumovirus.<br>• J21.8: Acute bronchiolitis due to other specified organisms.<br>• J21.9: Acute bronchiolitis, unspecified.<br>For the secondary analysis, the relative proportion of these subclasses within all J21-coded hospitalisations were examined. Of note, J21.1, J21.8 and J21.9 were not included in identifying RSV-related hospitalisations for the primary analysis of hospitalisation rates.	Children aged <24 months

Methods S1. Methods and limitations

Hospitalisation data
Hospitalisation data from January 2016 to December 2022 were obtained from the Australian Institute of Health and Welfare (AIHW) National Hospital Morbidity Database. Primary estimates of RSV-coded hospitalisation rates were based on the pre-COVID-19 period (up to December 2019) due to the substantial pandemic-related disruption to RSV epidemiology. Data from 2020–2022 were used for sensitivity analyses to assess differences in post-pandemic age-distribution patterns.

This study reports overall hospitalisation rates for all Australians, including Aboriginal or Torres Strait Islander peoples. However, age-stratified data for Aboriginal and Torres Strait Islander adults aged ≥65 years were unavailable due to the small number and the need to protect against potential re-identification. Consequently, while broad aggregated age groups (i.e. adults ≥60 and ≥65 years) included the total Australian population, narrow-band age-specific analyses within older adults (e.g. 65-69 years and 70-75 years) were restricted to non-Indigenous Australians only.

National Notifiable Diseases Surveillance System (NNDSS)
Laboratory-confirmed RSV cases became a nationally notifiable disease in July 2021, however jurisdictions commenced reporting of complete data at different times over 2021 and 2022 (e.g., from July 2021 in WA; from Sep 2022 in NSW). As such, this report conducted analyses on the 2023 data, the first year of complete jurisdictional reporting nationwide. Of note, NNDSS is based on case surveillance and the number of tests performed is unknown.

Data from 2024 were not included in analyses due to the potential effects of jurisdictional infant immunisation program roll-outs on notification patterns.

Rate calculations for hospitalisations and notifications
To calculate hospitalisation and notification rates, mid-year Australian resident population estimates from the Australian Bureau of Statistics (ABS) were matched to the corresponding admission and notification years.

RSV seasonality
RSV seasonality is less pronounced in some parts of Australia, particularly in northern regions such as the Northern Territory and the Northern tropical region of Western Australia. However, prior to the COVID-19 pandemic, most areas exhibited a distinct seasonal pattern. Considering national trends, RSV in-season was defined as April–September, with all other months classified as inter-season.

Missing data
Twenty-six of 128,122 notified cases (0.02%) lacked recorded ages and were excluded from age-specific rate calculations. Given this negligible fraction, their exclusion is unlikely to bias the observed age distribution. No missing data impacting the analysis were identified within the RSV-related hospitalisation records.

Age grouping
Age groupings were established to align with key immunisation policies and programs outlined in the *Australian Immunisation Handbook*, while ensuring comparability with existing literature.

Ethics
This study was approved by the Sydney Children's Hospitals Network Human Research Ethics Committee (2024/ETH01925)

Limitations
This study did not evaluate specific subpopulations potentially at greater risk of severe RSV disease—such as individuals with comorbidities, specific occupational groups, or Aboriginal and Torres Strait Islander peoples.

Because our estimates are intended to reflect the broad national average, they do not capture the distinct disease burden variations or inequities present within these specific populations. These priority populations warrant targeted future research.

Table S2. STROBE Statement—Checklist of items that should be included in reports of *cohort studies*

	Item No	Recommendation	Page number
**Title and abstract**	1	(*a*) Indicate the study's design with a commonly used term in the title or the abstract	Title page
		(*b*) Provide in the abstract an informative and balanced summary of what was done and what was found	N/A (Research letter)
**Introduction**			
Background/rationale	2	Explain the scientific background and rationale for the investigation being reported	p1
Objectives	3	State specific objectives, including any prespecified hypotheses	p1
**Methods**			
Study design	4	Present key elements of study design early in the paper	p1
Setting	5	Describe the setting, locations, and relevant dates, including periods of recruitment, exposure, follow-up, and data collection	p1
Participants	6	(*a*) Give the eligibility criteria, and the sources and methods of selection of participants. Describe methods of follow-up	p1
		(*b*) For matched studies, give matching criteria and number of exposed and unexposed	n/a
Variables	7	Clearly define all outcomes, exposures, predictors, potential confounders, and effect modifiers. Give diagnostic criteria, if applicable	p1
Data sources/ measurement	8*	For each variable of interest, give sources of data and details of methods of assessment (measurement). Describe comparability of assessment methods if there is more than one group	p1 & supporting information
Bias	9	Describe any efforts to address potential sources of bias	p2-p3 (highlighting potential bias in the Results and Discussion sections)
Study size	10	Explain how the study size was arrived at	n/a

	Item No	Recommendation	Page number
Quantitative variables	11	Explain how quantitative variables were handled in the analyses. If applicable, describe which groupings were chosen and why	p1 & supporting information
Statistical methods	12	(*a*) Describe all statistical methods, including those used to control for confounding	p1 & supporting information
		(*b*) Describe any methods used to examine subgroups and interactions	n/a
		(*c*) Explain how missing data were addressed	Supporting information
		(*d*) If applicable, explain how loss to follow-up was addressed	n/a
		(*e*) Describe any sensitivity analyses	p2
**Results**			
Participants	13*	(a) Report numbers of individuals at each stage of study—eg numbers potentially eligible, examined for eligibility, confirmed eligible, included in the study, completing follow-up, and analysed	p2
		(b) Give reasons for non-participation at each stage	n/a
		(c) Consider use of a flow diagram	n/a
Descriptive data	14*	(a) Give characteristics of study participants (eg demographic, clinical, social) and information on exposures and potential confounders	p2
		(b) Indicate number of participants with missing data for each variable of interest	n/a
		(c) Summarise follow-up time (eg, average and total amount)	n/a
Outcome data	15*	Report numbers of outcome events or summary measures over time	p2
Main results	16	(*a*) Give unadjusted estimates and, if applicable, confounder-adjusted estimates and their precision (eg, 95% confidence interval). Make clear which confounders were adjusted for and why they were included	p2, Supporting information
		(*b*) Report category boundaries when continuous variables were categorized	n/a

	Item No	Recommendation	Page number
		(*c*) If relevant, consider translating estimates of relative risk into absolute risk for a meaningful time period	n/a
Other analyses	17	Report other analyses done—eg analyses of subgroups and interactions, and sensitivity analyses	p2, Supporting information
**Discussion**			
Key results	18	Summarise key results with reference to study objectives	p2-p3
Limitations	19	Discuss limitations of the study, taking into account sources of potential bias or imprecision. Discuss both direction and magnitude of any potential bias	p3
Interpretation	20	Give a cautious overall interpretation of results considering objectives, limitations, multiplicity of analyses, results from similar studies, and other relevant evidence	p3
Generalisability	21	Discuss the generalisability (external validity) of the study results	p3, supporting information
**Other information**			
Funding	22	Give the source of funding and the role of the funders for the present study and, if applicable, for the original study on which the present article is based	Title page

*Give information separately for exposed and unexposed groups.

Note: An Explanation and Elaboration article discusses each checklist item and gives methodological background and published examples of transparent reporting. The STROBE checklist is best used in conjunction with this article (freely available on the Web sites of PLoS Medicine at http://www.plosmedicine.org/, Annals of Internal Medicine at http://www.annals.org/, and Epidemiology at http://www.epidem.com/). Information on the STROBE Initiative is available at http://www.strobe-statement.org.

Table S3. Age-specific RSV-related hospitalisation rates by year

Age groups	2016	2017	2018	2019
<3 months	3,771	3,856	4,007	4,436
3-5 months	2,040	2,056	2,168	2,452
6-11 months	1,155	1,222	1,298	1,512
12-23 months	700	667	725	936
24-59 months	131	130	136	190
5-19 years	10	10	9	13
20-49 years	5	7	7	8
50-59 years	15	20	23	25
60-64 years	28	36	43	45
65-69 years*	38	56	58	60
70-74 years*	56	81	90	93
75-79 years*	82	125	126	146
≥80 years*	148	242	285	314
Aggregated age groups				
<6 months	2,905	2,956	3,087	3,444
<12 months	2,030	2,089	2,192	2,478
<24 months	1,372	1,363	1,453	1,706
≥60 years†	65	100	113	123
≥65 years†	79	123	138	150
All ages†	60	68	72	83

* Age-specific analysis for people aged ≥65 years includes only people who did not identify as Aboriginal or Torres Strait Islander. Age-stratified hospitalisation data for Aboriginal and Torres Strait Islander peoples aged ≥65 years were not supplied to the authors due to small numbers and protection against individual identification. † Aboriginal and Torres Strait Islander peoples aged ≥65 years are included in these aggregated age groups.

Table S4. In-season and inter-season RSV-related hospitalisation rates and proportions of annual rate, 2016-2019.

Age group	Rate per 100,000 (% of annual rate)	
	In-Season	Inter-season
<6 months	2,488 (80.4)	607 (19.6)
<12 months	1,732 (78.9)	463 (21.1)
<24 months	1,149 (78.1)	322 (21.9)

Figure S1. Hospitalisations with a principal diagnosis of acute bronchiolitis (J21) by causative organisms, 2016-2019.

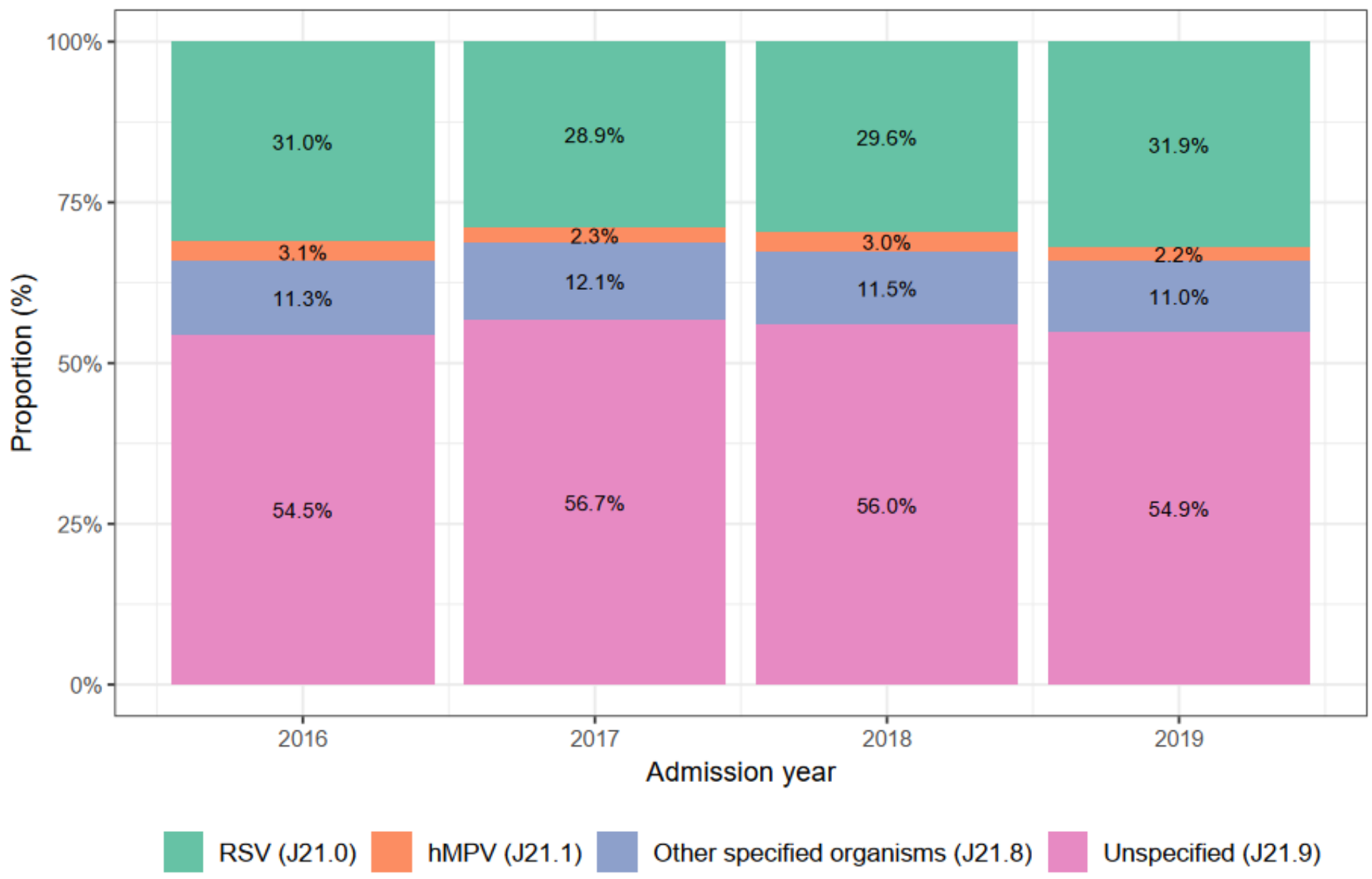

Figure S2. RSV-coded hospitalisation rates: 2016–2019 (pre-COVID-19) and 2020–2022.

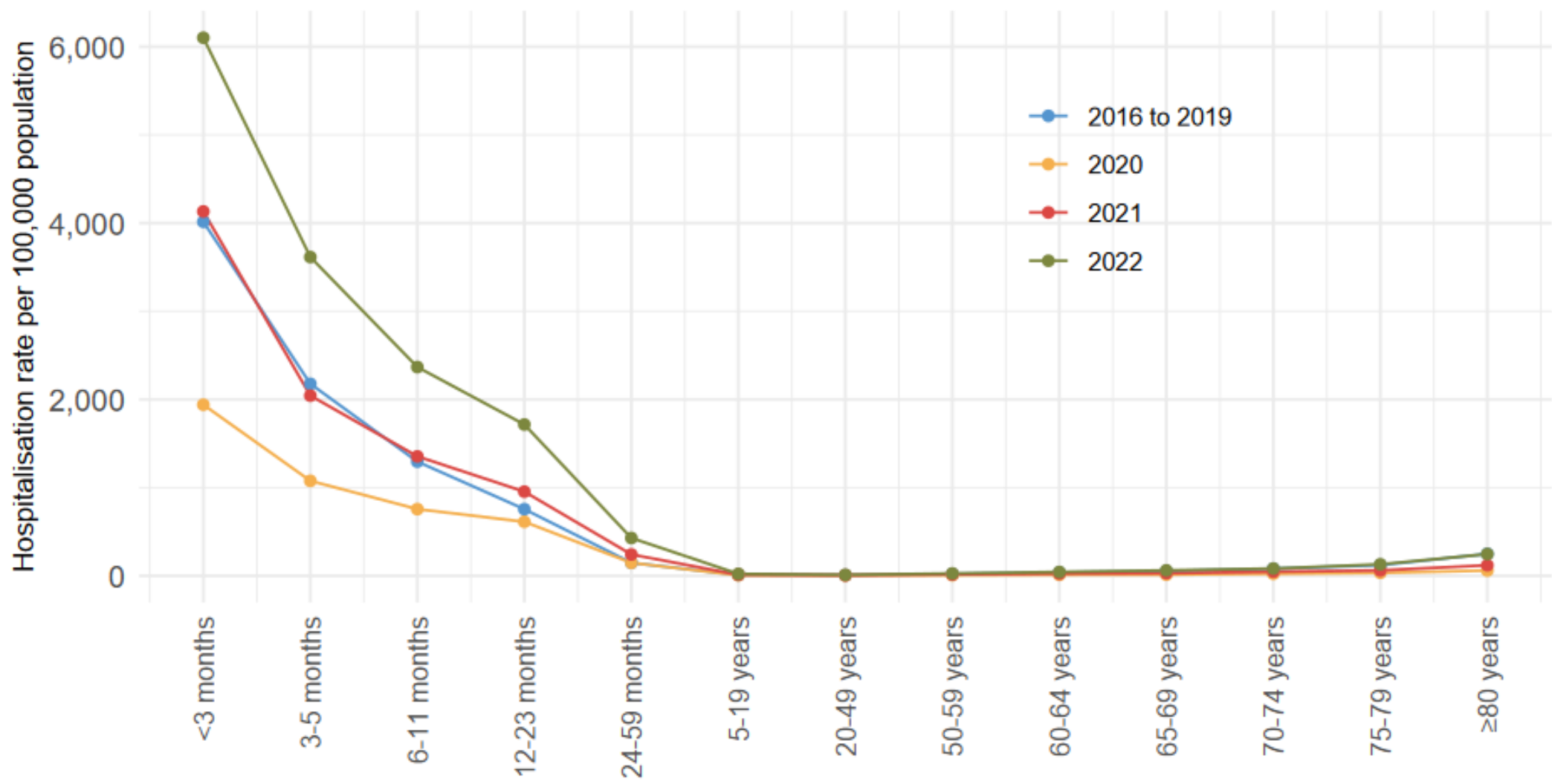

Supplement: Supplementary file 1 [file MJA2-224-0-s001.pdf]
